# *N*-Acetylglutaminoyl-*S*-farnesyl-l-cysteine (SIG-1191): an anti-inflammatory molecule that increases the expression of the aquaglyceroporin, aquaporin-3, in human keratinocytes

**DOI:** 10.1007/s00403-016-1708-x

**Published:** 2016-12-17

**Authors:** José R. Fernández, Corey Webb, Karl Rouzard, Michael Voronkov, Kristen L. Huber, Jeffry B. Stock, Maxwell Stock, Joel S. Gordon, Eduardo Perez

**Affiliations:** 1 Signum Dermalogix, 133 Wall Street, Princeton, NJ 08540 USA; 2 Department of Molecular Biology, Princeton University, Princeton, NJ USA

**Keywords:** Aquaglyceroporin-3, Skin hydration, Isoprenylcysteine, Inflammation

## Abstract

Isoprenylcysteine (IPC) small molecules were discovered as signal transduction modulating compounds ~25 years ago. More recently, IPC molecules have demonstrated antioxidant and anti-inflammatory properties in a variety of dermal cells as well as antimicrobial activity, representing a novel class of compounds to ameliorate skin conditions and disease. Here, we demonstrate a new IPC compound, *N*-acetylglutaminoyl-*S*-farnesyl-l-cysteine (SIG-1191), which inhibits UVB-induced inflammation blocking pro-inflammatory cytokine interleukin-6 (IL-6) and tumor necrosis factor alpha (TNF-α) production. To investigate further the previously reported hydrating potential of IPC compounds, SIG-1191 was tested for its ability to modulate aquaporin expression. Specifically, aquaporin 3 (AQP3) the most abundant aquaporin found in skin has been reported to play a key role in skin hydration, elasticity and barrier repair. Results show here for the first time that SIG-1191 increases AQP3 expression in both cultured normal human epidermal keratinocytes as well as when applied topically in a three-dimensional (3D) reconstructed human skin equivalent. Additionally, SIG-1191 dose dependently increased AQP3 protein levels, as determined by specific antibody staining, in the epidermis of the 3D skin equivalents. To begin to elucidate which signaling pathways SIG-1191 may be modulating to increase AQP3 levels, we used several pharmacological pathway inhibitors and determined that AQP3 expression is mediated by the Mitogen-activated protein kinase/Extracellular signal-regulated kinase kinase (MEK) pathway. Altogether, these data suggest SIG-1191 represents a new IPC derivative with anti-inflammatory activity that may also promote increased skin hydration based on its ability to increase AQP3 levels.

## Introduction

The addition of farnesyl (15-carbon side chain) or geranylgeranyl (20-carbon side chain) isoprenoids is essential to the membrane targeting of heterotrimeric and small G proteins that mediate receptor signaling in eukaryotic cells. This post-translational modification occurs within a CAAX-tail motif, where the isoprenoid is attached to a cysteine residue via a thioether bond [48]. Isoprenylcysteine (IPC) analogs contain the same 15- or 20-carbon side chain mimicking the C terminus of processed CAAX proteins [52]. IPC analogs upon cellular uptake affect signaling activities by several mechanisms. First, through insertion into the membrane, they compete with isoprenoid groups for prenyl-binding sites on membrane-associated proteins [11, 30, 34, 43]. In addition, IPC analogs modulate signal transduction by inhibiting heterotrimeric G protein formation [12, 17] and by binding and activating peroxisome proliferator-activated receptor gamma (PPARγ) [6].

IPC analogs have been initially identified as a novel class of topical anti-inflammatory compounds [15, 16, 19]. In vitro studies have shown IPC compounds to be effective down modulators of inflammatory responses in platelets, macrophages and neutrophils [25, 30, 41, 50]. IPC derivatives were shown to inhibit pro-inflammatory TNF-α stimulation of vascular cell adhesion molecule-1 (VCAM-1) by modulating small G protein Rac1 activity [3, 39] and suppressing purinergic receptor (a G protein-coupled receptor—GPCR)-mediated IL-8, monocyte chemotactic protein-1 (CCL2) and growth-regulated oncogene α (CXCL1) production [1]. Recent studies demonstrate IPC analogs also downregulate non-G protein-mediated inflammation in human epidermal keratinocytes, dermal fibroblasts and peripheral blood mononuclear cells by abrogating toll-like receptor 2 (TLR2), toll-like receptor 4 (TLR4) and T cell receptor (TCR) signaling [14]. Furthermore, IPC analogs have also been shown to inhibit ultraviolet A (UVA), ultraviolet B (UVB), phorbol 12-myristate 13-acetate (TPA) and bacteria-induced pro-inflammatory cytokine release [14, 15, 19] in skin cells, highlighting the effectiveness of this class of compounds in blocking cutaneous inflammation. Interestingly, we have also shown that in a human use study, IPCs have the potential to enhance skin hydration [16].

Aquaporins (AQPs) are a family of highly conserved transmembrane proteins that act primarily as water-selective pores, facilitating the transport of water across cell membranes [10]. Thirteen mammalian AQPs have been identified over the last 23 years with differing functions dependent on localization [23]. To date, AQP3, AQP9 and AQP10, called aqua-glycoporins because they transport water, glycerol and possibly other small solutes, have been found in skin [28, 42]. AQP3, the most abundant AQP found in skin, is located in the basal, suprabasal and stratum corneum layers [2, 18, 28, 33, 51] of the epidermis at the cell periphery [47]. The critical role(s) AQP3 plays in skin hydration were elucidated using AQP3 knockout mice. These studies demonstrated mice lacking AQP3 had reduced stratum corneum hydration and elasticity, exhibiting delayed barrier recovery and wound healing [21]. In addition, altered AQP3 expression and production have also been linked to several skin diseases such as eczema [9, 38], psoriasis, non-melanoma skin cancers [23, 31, 44, 52] and vitiligo [29]. Recently, AQP3 has also been shown to play a protective role in UV-induced apoptosis in fibroblasts [54] and has also been suggested to be a participating factor in skin aging [45]. Thus, AQP3 is a key player in the skin and identifying compounds that modulate its activity could yield new therapeutic actives for drug development and skin care.

**Fig. 1:**
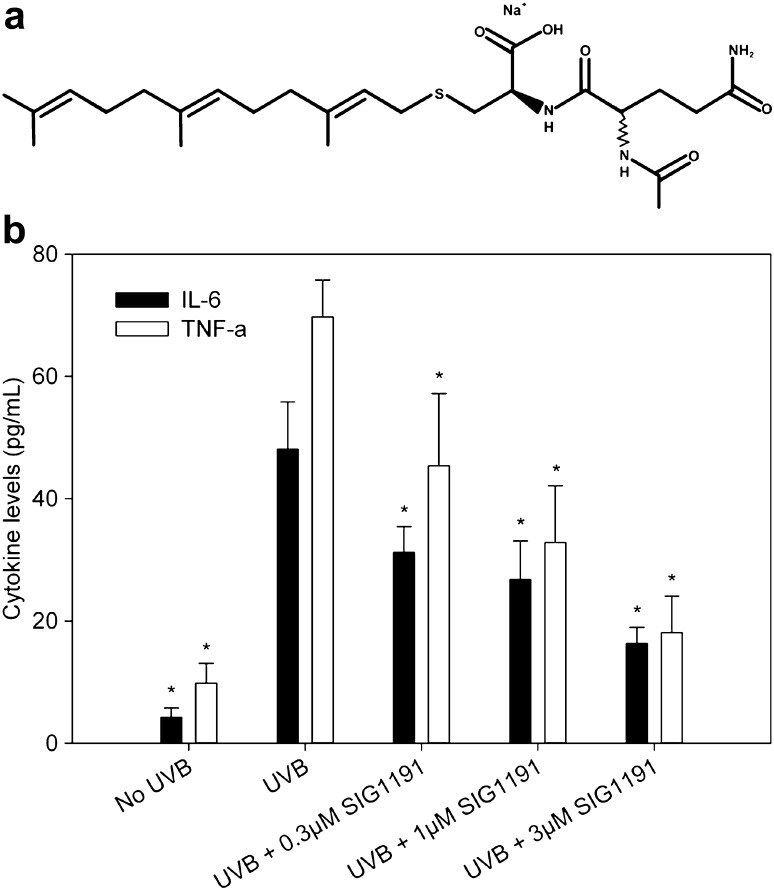
SIG-1191 has anti-inflammatory activity blocking UVB pro-inflammatory cytokine production. **a** Chemical structure of monosodium salt of *N*-acetylglutaminoyl-*S*-farnesyl-l-cysteine (SIG-1191) **b** Cells were incubated with SIG-1191 for 6 h, washed and irradiated in PBS with 25 mJ/cm^2^ UVB and later incubated without SIG-1191 in supplement-depleted media for 24 h. Pro-inflammatory cytokines (IL-6 and TNF-α) were measured from media supernatants by ELISA. The data represent the mean ± SEM of cumulative from three independent experiments. *p < 0.05 indicates a statistically significant difference compared to UVB-only irradiated cells

Here, we report that IPC analog *N*-acetylglutaminoyl-*S*-farnesyl-l-cysteine (SIG-1191) (Fig. 1a) in which a glutamine is thioacetylated to the cysteine residue possesses anti-inflammatory properties by inhibiting UV-induced pro-inflammatory mediators in human keratinocytes, similar to previous IPC compounds [15, 16]. Furthermore, we demonstrate for the first time that SIG-1191 upregulates AQP3 gene expression in monolayer keratinocytes and both gene expression and protein production in reconstructed three-dimensional human skin cultured at the air–liquid interface. Using specific pathway inhibitors, induction of AQP3 expression is demonstrated to be mediated by MAPK kinase (MEK)-dependent pathways. Altogether, these results suggest SIG-1191 may be effective in boosting skin hydration, elasticity and barrier recovery through its ability to increase AQP3 levels in addition to reducing inflammation.

## Materials and methods

### Reagents

SIG-1191 (*N*-acetylglutaminoyl-*S*-farnesyl-l-cysteine) was synthesized according to the methods described in US patent application US 12/616,781. The monosodium salt of SIG-1191 was used for all assay treatments (Fig. 1a). All chemicals were analyzed by LC/MS (Agilent 1100), ^1^H and ^13^C NMR (500 and 125 MHz, Bruker) for structural identity and confirmed to be >93% pure by analytical HPLC (Agilent 1200; Santa Clara, CA).

### Monolayer NHEK cell and 3D reconstructed human skin tissue culture

Normal human epidermal keratinocytes (NHEKs) from neonatal donors were obtained from ThermoFisher (Carlsbad, CA). Cells were cultured until the second passage and seeded on six-well plates for 24 h before treatments with EpiLife^®^ media supplemented with keratinocyte growth supplement and 60 µM calcium (ThermoFisher; Carlsbad, CA).

The EpiDerm-FT™ skin cultures, reconstructed from cells of human origin, were purchased as preserved culture inserts from MatTek Corp. (Ashland, MA). They were cultured and acclimated at the air–liquid interface at 37 °C and 5% CO_2_ for 24 h in six-well plates. SIG-1191 formulated in water was single administered by topical application (25 µL) and incubated for 24 h.

### Gene expression

NHEK cell monolayers and EpiDerm-FT™ tissues were treated for 24 h. Harvested cells and biopsy punches (8 mm) were obtained from EpiDerm-FT™ cultures after treatments and homogenized using a Dounce homogenizer (1 mL capacity). Total RNA was extracted using the RNAqueous kit (Ambion^®^) and cDNA was obtained using the High-Capacity RNA-to-cDNA kit (Applied Biosystems). Quantitative PCR (qPCR) was performed using the TaqMan^®^ Fast Advanced Master Mix (Applied Biosystems) and specific TaqMan^®^-probes human gene primers for AQP3, AQP9 and GAPDH to calculate the relative gene fold expression change per treatment. Gene expression analysis was performed using the comparative Ct method (2–[delta][delta] Ct) approach by comparing the Ct values of the treated samples with the untreated samples and normalized to GAPDH gene expression as endogenous housekeeping gene.

### Histology

After 24-h treatments, EpiDerm-FT™ cultures were fixed in 10% formalin and later preserved by paraffin embedding. Sections (5 µm) were stained with hematoxylin and eosin (H&E) for evaluation of tissue morphology. Immunohistochemistry (IHC) was performed using anti-aquaporin-3 (AbCam; Cambridge, UK) and anti-keratin-10 (EMD Millipore; Billerica, MA). Secondary antibodies used were anti-rabbit-Alexa Fluor-488 and anti-mouse-Alexa Fluor-594 (Jackson Immuno Research Labs; West Grove, PA). Antibody specificity was confirmed by staining tissues with secondary antibodies only. Stained sections were viewed using an Olympus BX-41 microscope (Center Valley, PA) equipped with CCD camera (Hamamatsu Photonics K.K.; Shizuoka Pref., Japan) and using MetaMorph^®^ software (Molecular Devices, Sunnyvale, CA).

### UVB radiation

Monolayer NHEKs were treated with SIG-1191 monosodium salt (0, 0.3, 1, 3 µM) for 6 h at 37 °C and 5% CO_2_ in depleted media (without growth factors). Later, treatment media was removed, cells were washed 3× with PBS and irradiated in PBS (phenol red-free) with 25 mJ/cm^2^ UVB using an external research irradiator (Daavlin Co, Bryan, OH) equipped with broadband UVB lamps (305 ± 12 nm). After irradiation, media was replaced with depleted media without SIG-1191 and incubated for 24 h at 37 °C and 5% CO_2_. After incubation, media supernatants were obtained and human IL-6 and TNFα were measured by standard sandwich ELISA following the recommended manufacturer’s protocol (BD Biosciences; San Jose, CA).

### Statistical analysis

Statistical significance was determined by one-way ANOVA followed by Bonferroni multiple comparisons test using *p* values less than 0.05 as a significant difference.

## Results

### SIG-1191 inhibits UVB-induced pro-inflammatory cytokine production

UVB-induced photoaging is mediated through production of inflammatory cytokines and MMPs induced through the activation of the MAP kinase and NFκB pathways [32]. The resulting induction of interleukin-6 (IL-6) and tumor necrosis factor alpha (TNF-α) also contributes to skin dryness [40]. We tested SIG-1191 to determine if it possessed similar characteristics to previously tested IPC analogs [15], specifically by decreasing UVB-induced pro-inflammatory cytokine release from cultured primary keratinocytes (NHEKs). Our results show SIG-1191 dose dependently inhibits IL-6 and TNF-α release with an IC_50_ = 1 µM (Fig. 1b).

### SIG-1191 increases AQP3 gene expression in epidermal cells

**Fig. 2:**
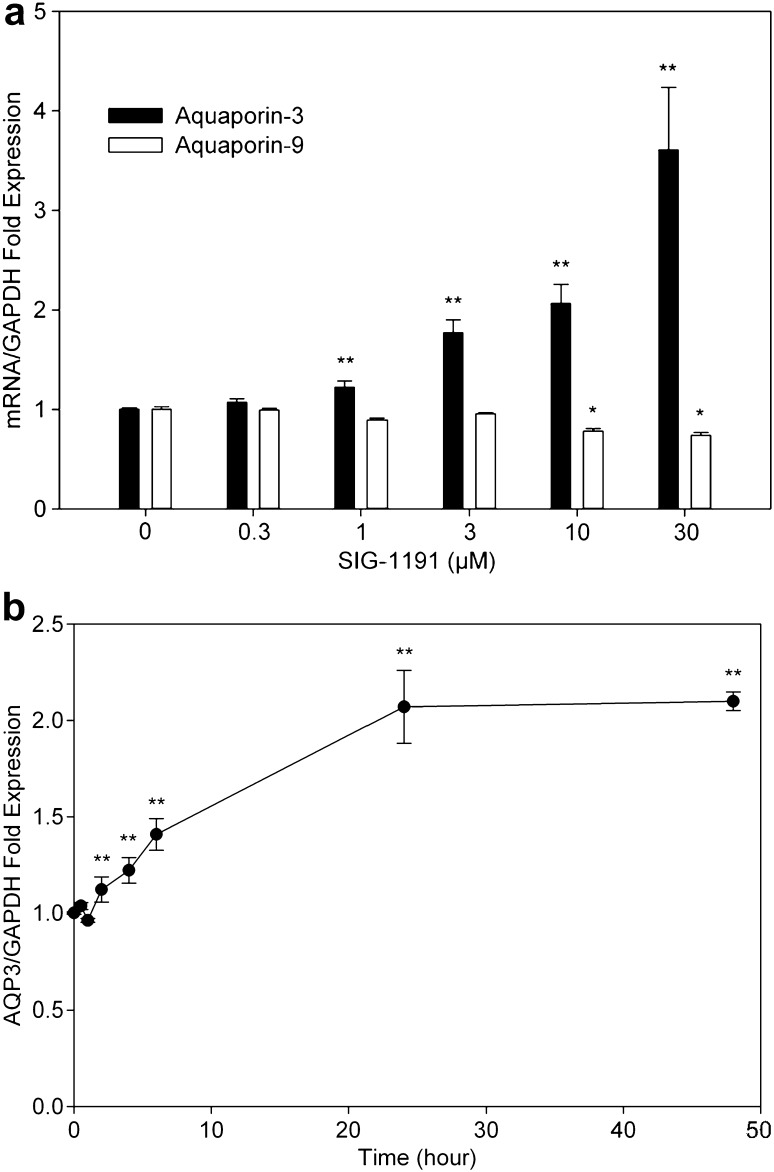
SIG-1191 increases AQP3 gene expression in a dose- and time-dependent manner. **a** NHEKs were treated with the indicated concentrations of SIG-1191 for 24 h. **b** Cells were treated with 10 µM SIG-1191 (2, 4, 6, 8, 24, 48 h) and harvested for gene expression analysis. The level of gene expression of aquaporins (AQP3, AQP9) was quantitated by qPCR normalizing to level of GAPDH the control housekeeping gene. The data represent the mean ± SEM of cumulative from three independent experiments. *p < 0.05; **p ≤ 0.01 indicates a statistically significant difference compared to untreated cells

UVB exposure damages skin water barrier function as determined by increased transepidermal water loss (TEWL) [24] and potentially downregulation of AQPs [26]. Since IPC compounds have been shown to improve hydration and skin firmness in a clinical study [16], we sought to determine if SIG-1191 could modulate the expression of human skin epidermal aquaporins (AQP3, AQP9). To investigate aquaporin gene expression activity, NHEK cells were treated with the indicated concentrations of SIG-1191 in media for 24 h. After incubation, cells were collected and gene expression was assessed by quantitative PCR (qPCR). After 24 h, SIG-1191 at ≥3 µM significantly increased AQP3 gene expression +216–609% (Fig. 2a). Conversely, SIG-1191 only at the highest concentrations tested (≥10 µM) had a slight, but significant decrease on AQP9 expression (30% reduction), an understandable result given that AQP9 expression in keratinocytes has been reported to be regulated in a different manner than that of AQP3 [49]. SIG-1191 significantly increased AQP3 gene expression after only 2 h of exposure and reaching maximum levels at 24–48 h (Fig. 2b). After 48-h treatments, the increased expression of AQP3 remained stable.

### Increase of AQP3 expression is mediated by the MEK pathway

**Fig. 3:**
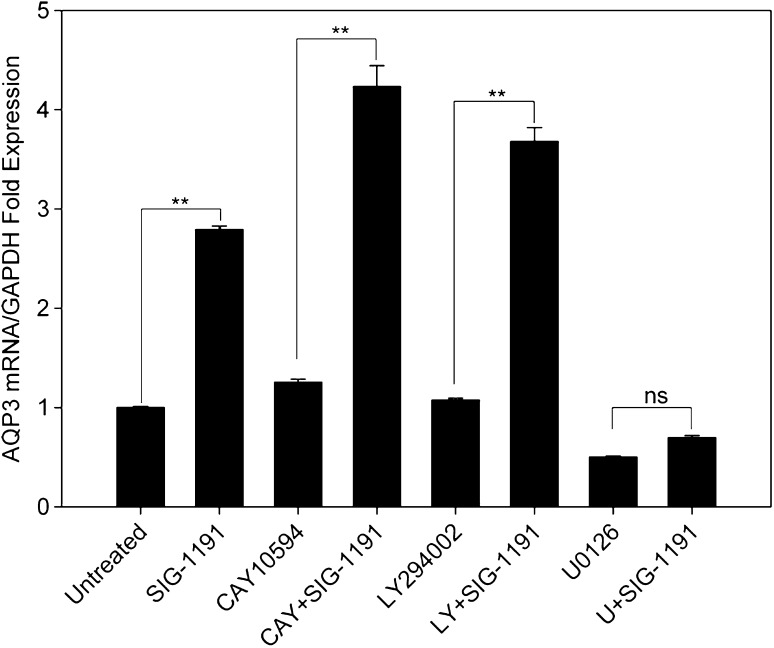
AQP3 gene expression is activated by the MEK pathway. NHEKs were treated with 10 µM SIG-1191 with and without the indicated pathway inhibitors (1 µM CAY10594 (CAY) a phospholipase D2 inhibitor; 10 µM LY294002 (LY) a phosphatidylinositol 3-kinase inhibitor; 10 µM U0126 (U) a MAPK/ERK kinase inhibitor) for 24 h and harvested for AQP3 mRNA levels. Gene expression of AQP3 was analyzed by qPCR normalized to GAPDH, a control housekeeping gene. The data represent the mean ± SEM of cumulative from three independent experiments. *p < 0.05; **p ≤ 0.01 indicates a statistically significant difference compared to untreated cells (*ns* not significant)

The mRNA levels of AQP3 in epidermal keratinocytes have been shown to be dependent on several signaling pathways including those that involve phosphatidylinositol 3-kinase and MAPK kinases [4, 9, 20, 27]. In addition, AQP3 has been shown to associate with phospholipase D2 (PLD2) to promote keratinocyte differentiation pathway [7]. Therefore, we used pharmacological agents that inhibit these specific signaling pathways to investigate which signal(s) is (are) used by SIG-1191 to modulate AQP3 expression. NHEKs were treated with phospholipase D2 (CAY10594), phosphatidylinositol 3-kinase (LY294002) and MAPK kinases (U0126) inhibitors and co-treated with SIG-1191 (10 µM) in media for 24 h. After incubation, cells were collected and AQP3 gene expression was assessed by qPCR. Results show that U0126 alone significantly reduced AQP3 mRNA levels compared to untreated cells. In addition, U0126 inhibits the SIG-1191-induced increase in AQP3 expression (Fig. 3), consistent with the involvement of MAPK kinase (MEK)-dependent signaling pathway in SIG-1191-induced AQP3 expression in NHEKs.

### SIG-1191 increases AQP3 gene and protein expression in a 3D human skin model

**Fig. 4:**
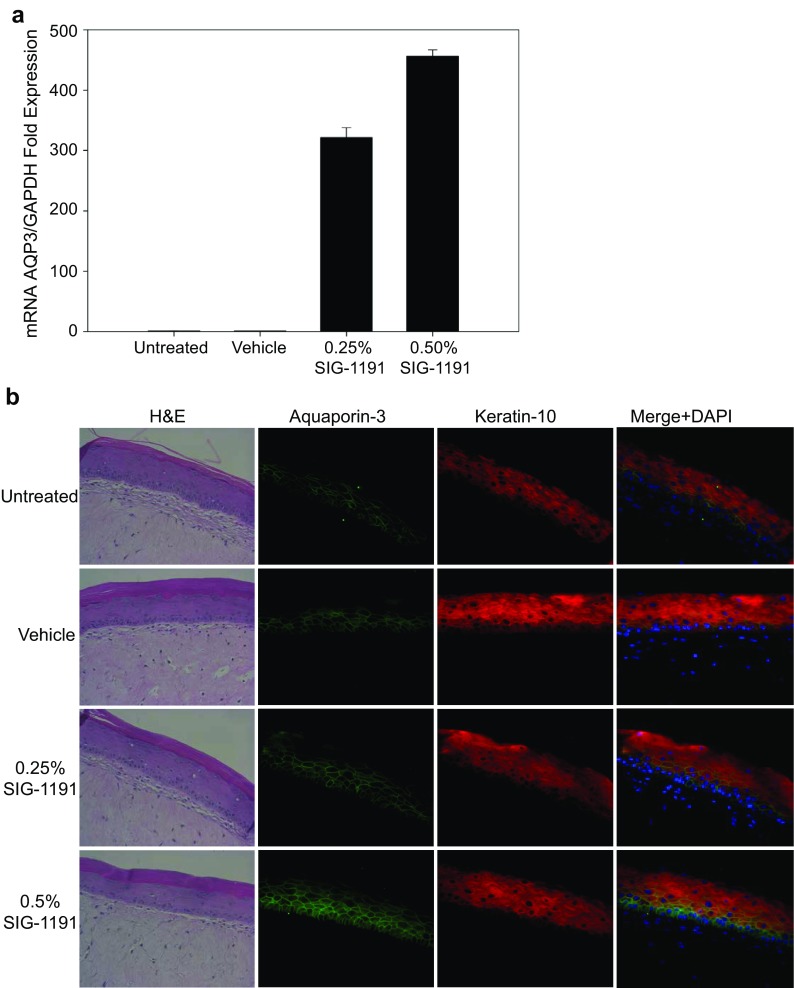
SIG-1191 increases AQP3 gene expression and protein levels in Reconstructed Human Epidermis (RHE). EpiDerm-FT™ air–liquid interface cultures were topically treated with 0.25–0.5% (w/v) of SIG-1191 for 24 h. **a** AQP3 gene expression was analyzed by qPCR normalized to GAPDH a control housekeeping gene. The data represent the mean ± SEM of a representative experiment. **b** Haematoxylin and eosin (H&E) staining and immunohistochemistry (IHC) of EpiDerm-FT™ tissues. Immunohistochemistry was performed with anti-aquaporin-3 (*green*), anti-keratin-10 (*red*), anti-rabbit Alexa-488, anti-mouseAlexa-594 antibodies. Sections were counterstained with 2-(4-amidinophenyl)-1H-indole-6-carboxamidine (DAPI). Merged micrograph shows overlaying K10 and AQP3 antibodies with DAPI staining. No background fluorescence was observed in the absence of the primary antibody (not shown). Original magnification: ×400

SIG-1191-induced AQP3 expression was validated using the full-thickness EpiDerm™ reconstructed human skin model (MatTek, Corp.) cultured at the air–liquid interface. Tissues were topically treated with SIG-1191 for 24 h and AQP3 mRNA accumulation was assessed by qPCR. SIG-1191 at 0.25–0.5% w/v in a dose-dependent manner significantly increased AQP3 gene expression +322- and 456-fold, respectively, after 24 h (Fig. 4a). Topical treatment of the reconstructed skin cultures showed no alteration of the morphology as revealed by H&E staining, nor was there any effect on the level of staining of suprabasal keratin-10 (K10) (Fig. 4b). AQP3 antibody staining localized to basal layer and partially to the lowest suprabasal layer, predominantly restricted to the cell periphery in untreated and vehicle-exposed cultures (Fig. 4b). Treatment with SIG-1191 increased in an apparent dose-dependent manner the intensity and distribution of AQP3 staining. For example, after treatment with SIG-1191 at 0.25%, AQP3 protein expression is visualized in the mid-suprabasal layers, while SIG-1191 applied at 0.5% increased overall intensity of staining, with AQP3 now seen throughout the cell cytoplasm and cell periphery staining detected in the suprabasal layers. This observation is emphasized by overlaying K10 and AQP3 antibody staining (Fig. 4b). Thus, when applied topically, SIG-1191 induces an increase in AQP3 mRNA expression and protein production.

## Discussion

In this study, we set to characterize the properties of *N*-acetylglutaminoyl-*S*-farnesyl-l-cysteine (SIG-1191), a novel IPC analog. The archetype of the farnesylated class of IPC molecules, *N*-acetyl-*S*-farnesyl-l-cysteine (AFC) was discovered ~25 years ago [50] and was subsequently shown to block chemically induced inflammation when applied topically in vivo [19]. Moreover, a different IPC farnesyl derivative called SIG-990 has also demonstrated anti-inflammatory activity and is being developed as a potential therapeutic for rosacea. Another farnesyl IPC, *S*-farnesylthiosalicylic acid has been shown to target inflammation in animal models of contact dermatitis and allergic inflammation [36, 37]. Building upon these results, we found that SIG-1191, similar to its IPC predecessors, effectively reduces inflammation as shown by the inhibition of UVB-induced IL-6 and TNF-α (Fig. 1b) production with greater potency than AFC in NHEKs (unpublished results). This suggests that the chemical modification of adding a glutamine residue to the IPC chemical backbone confers additional anti-inflammatory activity.

Aquaporins (AQPs) are pore-like passive channel transporters of water or glycerol that are widely expressed. AQP3 and AQP9 are expressed by human skin and have been shown to contribute to stratum corneum (SC) hydration and glycerol transport [13]. AQP3-null mice showed high-frequency skin conductance and ^3^H_2_O partitioning was reduced compared to wild-type animals, suggesting the critical role of AQP3 in SC hydration [21]. AQP3 is mostly expressed at the basal layer of the epidermis [46] and its expression is downregulated by UVB irradiation [26, 46]. It is present in both viable and stratum corneum layers of the epidermis [28], thus suggesting the functional role in water transport. Interestingly, the water loss prevention of the stratum corneum is linked to the specific expression of AQP3 between the dermis and basal layers of the epidermis, thus operating as a “water-clamp” to prevent water loss gradient from the dermis to the stratum corneum [47]. Since we have previously demonstrated that IPCs enhance skin hydration in human use studies [16] we utilized SIG-1191 to elucidate the possible molecular basis for this enhancement of skin hydration. To this end, we selected AQP3 and AQP9 as initial targets. Our results show primary keratinocytes exposed to ≥3 µM SIG-1191 after 24 h induce an increase in AQP3 gene expression through activation of a specific signaling pathway with a minimal decrease on AQP9 expression (Fig. 2). When topically applied on human reconstructed skin (EpiDerm-FT™ model), SIG-1191 induced AQP3 mRNA overexpression (Fig. 4a) and via immunohistological analysis, AQP3 was visibly overexpressed 24 h after treatment of treatment with SIG-1191 (Fig. 4b).

AQP3 expression is upregulated by both nuclear and membrane receptor activation [53]. Another commonly used skin care compound, retinoic acid has been shown to increase AQP3 and also slightly decrease AQP9 gene and protein expression via the nuclear retinoic acid receptor subtype gamma (RARγ) [5, 49]. The involvement of this receptor in any potential SIG-1191 AQP3-mediated enhancement of skin hydration is unlikely since retinoids are associated with skin drying. The upregulation of AQP3 is more likely part of the barrier repair response secondary to the drying effects of retinoic acid. Other studies demonstrate the nuclear PPARγ receptor stimulates AQP3 expression in keratinocytes [27]. Previous studies showed IPCs were ligands for PPARγ [6]. However, reporter assays with SIG-1191 revealed it was not a PPARγ activator (unpublished results).

Since, AQP3 gene expression is regulated by the activation of transcription factors by different membrane receptor-activated signaling pathways [4, 7, 9, 20], using specific inhibitors to each of the known pathways, we found SIG-1191 controls AQP3 gene expression through the MAPK/ERK kinase pathway (Fig. 3). Activation of this pathway has been demonstrated to be critical for skin hydration and re-epithelialization during wound healing [9, 22, 35]. IPC derivatives shown to modulate either GPCR or toll-like receptor (TLR)-activated pathways are not known to regulate AQP3 expression or to generally signal through the MEK pathway. The small G protein Ras, which has been previously shown to be modulated by IPCs, signals via the MAPK kinase pathway [41]. Thus, the possibility of this pathway remains open to be modulated by IPC compounds, until it is determined which Ras-dependent pathway plays a role in regulating AQP3 expression. It should be noted that IPC analogs have only been shown to downregulate Ras signaling and increased expression of AQP3 would require that SIG-1191 upregulates Ras signaling. Specifically, the EGF receptor, which activates the MEK pathway through Ras, is known to upregulate AQP3 expression in keratinocytes [9]. Therefore, it is possible that the EGF receptor is also IPC sensitive or that SIG-1191 modulates GPCR and/or TLR-activated pathways that impinge on MEK signaling.
